# Nutritional Strategies for Managing Iron Deficiency in Adolescents: Approaches to a Challenging but Common Problem

**DOI:** 10.1016/j.advnut.2024.100215

**Published:** 2024-03-29

**Authors:** Clay T Cohen, Jacquelyn M Powers

**Affiliations:** Division of Hematology/Oncology, Department of Pediatrics, Baylor College of Medicine and Texas Children’s Cancer and Hematology Center, Texas Children’s Hospital, Houston, TX, United States

**Keywords:** Iron deficiency, adolescent medicine, iron, anemia

## Abstract

Iron deficiency (ID) is a common and challenging problem in adolescence. In order to prevent, recognize, and treat ID in this age range, it is critical to understand the recommended daily intake of iron in relation to an adolescent’s activity, dietary habits, and basal iron losses. Adolescents following vegetarian or vegan diets exclusively rely on plant-based, nonheme iron, which has decreased bioavailability compared with heme iron and requires increased total iron intake. Individuals with disordered eating habits, excessive menstrual blood loss, and certain chronic health conditions (including inflammatory bowel disease and heart failure) are at high risk of ID and the development of symptomatic iron deficiency anemia (IDA). Adolescent athletes and those with sleep and movement disorders may also be more sensitive to changes in iron status. Iron deficiency is typically treated with oral iron supplementation. To maximize iron absorption, oral iron should be administered no more than once daily, ideally in the morning, while avoiding foods and drinks that inhibit iron absorption. Oral iron therapy should be provided for ≥3 mo in the setting of ID to reach a ferritin of 20 ng/mL before discontinuation. Intravenous iron is being increasingly used in this population and has demonstrated efficacy and safety in adolescents. It should be considered in those with persistent ID despite a course of oral iron, severe and/or symptomatic IDA, and chronic inflammatory conditions characterized by decreased gastrointestinal iron absorption.

**Statement of Significance**

Adolescence is a high-risk period for the development of iron deficiency, which often goes unrecognized. We provide a comprehensive review of the unique dietary requirements, highest-risk groups, and necessary treatment approaches for iron deficiency in adolescents.

## Introduction

Adolescence is characterized by rapid mental, physical, and social growth and development. Along with such growth comes increased iron demands due to expanding muscle mass, blood volume, increased hemoglobin concentration, and enzyme production. In menstruating individuals, onset of menarche results in additional iron loss. Dietary habits among this age group often result in low-iron intake and, at times, disordered eating, resulting in insufficient iron intake to maintain normal iron balance. Other risk factors that place additional teens at risk of iron deficiency (ID) include illness, high-intensity training in athletes, and blood donation. Thus, adolescence is a vulnerable time for the development of ID. Iron deficiency with or without anemia in adolescents can manifest as poor concentration, fatigue, and decreased cognition, resulting in poor school performance and a reduction in overall perceived quality of life across physical, mental, and social domains [[1], [2], [3], [4], [5], [6]]. Here, we review recommended dietary iron intake and sources for adolescents, those most at risk of becoming iron deficient, as well as optimal strategies for iron supplementation.

### Dietary considerations for adolescents

The recommended dietary allowance (RDA) for males and females in early adolescence (aged 9–13 y) is 8 mg iron/d. For the 14- to 18-y-olds, this increases to 11 mg/d and 15 mg/d in males and females, respectively. During individual growth spurts, demands may increase by 1 mg/d to 3 mg/d [7]. Despite these increased demands, the diets of many US adolescents are deficient in vitamins, minerals, and macro and micronutrients, including iron. Common habits include frequent snacking on energy-dense foods and beverages, skipping meals, and dieting [8]. These unhealthy eating habits contribute to a number of health-disorders, ranging from malnourishment to obesity.

It is worth noting that the dietary reference intake for iron was published in 2001 and estimated based on assumptions that may not reflect current adolescents. One assumption was that girls aged <14 y were premenarchal and thus that the increased iron RDA to account for menstrual blood loss begins in females aged 14 y. A 2018 study, however, demonstrated that the median age of menarche was 12.25 y [9]. The RDA of 8 mg iron/d for females <14 y is, therefore, likely to be an underestimate. Additionally, the estimated basal iron loss for males is based on a 1968 study by Green et al. [10], which may not reflect modern adolescent basal iron losses.

### Incorporation of iron-rich foods into diet

An emphasis on the incorporation of iron-rich foods into their daily diet is critical for adolescents to achieve their RDA, and even those adolescents receiving iron supplementation benefit from dietary changes to prevent recurrence of ID after their treatment course is complete. To maintain a normal iron balance, diets should include a variety of iron bioavailable foods. Dietary iron comes in 2 forms: heme and nonheme. Heme iron is contained in foods from animal sources and has higher bioavailability and absorption relative to nonheme iron (Table 1). When consumed together, heme iron also enhances the absorption of nonheme iron. Consistent intake of heme iron is the most effective way to ensure appropriate iron absorption and iron balance.

**TABLE 1:** Dietary sources of iron (heme and nonheme forms) and inhibitors of iron absorption

Heme iron	Nonheme iron	Inhibitors of iron absorption
Beef	Fortified breakfast cereals	Calcium-rich foods (dairy, cow milk)
Chicken	Beans (white, black, kidney)	Coffee
Liver (chicken or beef)	Spinach	Green and black tea
Mollusks	Iron-enriched bread and rice	High phytates (grains, legumes, walnuts)
Oysters	Lentils	Oxalic acid (rhubarb, chocolate)
Clams	Peas	Egg protein
Veal	Nuts	Soy products
Fish (including canned sardines)	Dried Fruits	

Foods that contain nonheme iron include iron-fortified cereals and breads, white and kidney beans, lentils, spinach, peas, nuts, and dried fruits. Absorption of plant-based, nonheme iron is enhanced by the presence of ascorbic acid (found in citrus fruits, strawberries, sweet peppers, tomatoes, and broccoli) [11]. Common inhibitors of nonheme iron absorption in the diet are phytates (present in legumes, rice, and grains), polyphenols (tea and coffee), soybean protein, and calcium [7,12]. Lean meat, seafood, and poultry contain both heme and nonheme iron.

## Adolescents Affected by ID

### Adolescents with low-iron or restricted diets

#### Vegetarian or vegan diets

Recently, the exclusion of animal and animal by-products from the diet has gained popularity among adolescents. There are various reasons for choosing a vegetarian or vegan diet, including cultural or religious practices. For some adolescents, however, this lifestyle change is made due to the desire to lose weight and may signal an underlying eating disorder [[13], [14], [15]]. Although many vegetarian and vegan diets may have a similar or higher total level of iron compared with nonvegetarian/vegan diets, the bioavailability of plant-based, nonheme iron (estimated at 10%) is lower than that of animal-based, heme iron (18% bioavailability) [7,16,17]. Moreover, in addition to directly providing heme iron, the consumption of meat and fish in a meal enhances the absorption of nonheme iron [18]. As a result, those with a vegetarian diet are more likely to have ID compared with those whose diet contains animal products [19]. Thus, the daily iron intake for individuals with vegan diets should be 1.8-times that of adolescents with omnivorous diets. A study on iron status of adults following vegetarian compared with omnivorous diets found that despite a higher mean daily intake of iron, vegans were found to have lower ferritin concentrations compared with healthy controls [20]. Adolescents with very strict vegetarianism may have even lower bioavailability diets (approaching 5% overall iron absorption), requiring even higher daily iron intake to meet physiologic requirements [7]. Individuals who follow vegetarian or vegan diets need to include many sources of nonheme iron to meet the same RDA.

#### Disordered eating

Adolescent individuals who develop a pathologic relationship to food and body image are at high risk of malnutrition and ID. The estimated prevalence of eating disorders ranges from 1%–22.7% in females and 0.3%–0.6% in males, with the mean age of onset of these disorders being 12.5 y [21,22]. The diagnosis of eating disorders in adolescent males may be delayed and underestimated compared with females due to their focus on lean mass and muscularity [23,24]. Although traditionally associated with non-Hispanic White adolescents of higher socioeconomic status, eating disorders are being increasingly recognized across racial, ethnic, gender, and socioeconomic groups [[25], [26], [27]]. Although less well recognized, overweight and obese adolescents may experience similar medical complications as underweight individuals with eating disorders, including psychiatric comorbidities and orthostatic instability [[28], [29], [30]].

The more common classifications of eating disorders include anorexia nervosa (AN), bulimia nervosa, and avoidant/restrictive food intake disorder (ARFID). AN is characterized by a purposeful restrictive caloric intake relative to physiologic energy demands because of a fear of gaining weight, often accompanied by an altered perception of one’s body weight or appearance. ID may be less common in female patients with AN due to resultant amenorrhea after reversion of their hypothalamic-pituitary-ovarian axis to the prepubertal state [31]. For those females with eating disorders who retain menstruation, however, ≤40% may have depleted iron stores [32]. Individuals treated for AN remain at high risk of developing ID after remission. In one study 85% of patients treated for AN had iron intake below recommended ranges 3 y after hospitalization [33]. This finding enforces the importance of ongoing nutritional counseling during and after an adolescent receives therapy for their eating disorder. Bulimia nervosa is characterized by repeated episodes of binge eating and compensatory behaviors to prevent secondary weight gain, frequently accompanied by insufficient oral iron intake [19]. Individuals with ARFID have disruptive eating patterns that result in a failure to meet their physiologic energy needs [34]. Those with ARFID have lower iron intake compared with healthy controls, with carbohydrates and bakery/potato products making up a large portion of their diet [21].

### Adolescents with menstrual blood loss

According to the American College of Obstetrics and Gynecology, the mean age of menarche in the United States is 12.5 y [35]. Onset of menarche increases basal iron losses. Abnormal uterine bleeding or heavy menstrual bleeding (HMB) further exacerbates such losses. The prevalence of HMB in adolescent females, defined as blood loss ≥80 mL (1.30 mg iron/d) per menstrual cycle or, more practically, blood loss that interferes with an individual’s quality of life, is between 34% and 50% [[36], [37], [38], [39]]. In comparison, females without HMB were estimated to lose 27.6 mL of blood per menstrual cycle (0.45 mg iron/d) (7). ID is present in ≤50% of adolescent females with HMB [40,41].

Although abnormal and HMB most commonly occurs in the first 2 to 3 y after menarche due to immaturity of the hypothalamic-pituitary-ovarian axis (38), an inherited bleeding disorder is present in ≤33% of adolescent females who are referred to a hematologist for evaluation of HMB, with von Willebrand Disease (VWD) being the most common [42]. Thyroid disease should be considered in a patient with other systemic symptoms. ID is common in all adolescent females with HMB undergoing evaluation for an inherited bleeding disorder. In one large cohort of adolescent females undergoing hematologic evaluation, the median ferritin value was <15 ng/mL in both those with and without a diagnosis of VWD [43]. Specific red flags on the clinical history consistent with HMB that would raise suspicion for an inherited bleeding disorder are listed in Table 2 [38]. Adolescent females who become pregnant are also at an increased risk of IDA [44].

**TABLE 2:** Clinical presentations of adolescents at risk for iron deficiency

Risk Factor	Clinical History	Recommended Approach
Menstrual blood loss	Heavy menstrual bleeding Cycles <21 d apart or lasting >7 d Changing product more than every 2 h “Flooding” Overflow bleeding onto clothing or bedding Clots quarter size or greater Fatigue, headache, reduced social or sports activity	Hemostatic evaluation for bleeding disorder (including evaluation for von Willebrand disease) Regulation of menstrual blood loss (via hormonal supplementation) Screen for anemia and iron deficiency Iron replacement therapy
Low-iron diet or disordered eating	Restricted eating pattern Avoidance of food groups Weight loss Excessive focus on appearance	Dietary counseling Psychosocial support services Screen for anemia and iron deficiency Iron replacement therapy
Chronic comorbid condition	Dietary intake and absorption Presence of concomitant inflammation Gastrointestinal symptoms and/or blood loss	Screen for anemia and iron deficiency with CBC and full iron panel (ferritin may be artificially elevated due to inflammation) Iron replacement therapy (oral vs. intravenous) depending on underlying condition

When screening for ID, assessing for anemia or microcytosis on a complete blood count alone is insufficient, as less than half of adolescent females with ID will have these hematologic findings [45]. Rather, serum ferritin should be obtained, with iron supplementation initiated for values <20 μg/L [46]. In addition to iron therapy, a key to improving the patient’s iron status is regulation of HMB, which can often be achieved with hormonal supplementation. Though data on the impact of hormone supplementation on iron status in adolescents is limited, a cross-sectional Tanzanian study of >1300 adolescent females that evaluated the impact of hormonal contraceptive use on iron status demonstrated that hormonal contraceptive use was negatively associated with ID, anemia, and ID anemia. The reduction in iron deficiency and IDA was sustained in those females who used hormones over a longer time period (>2 y) [47]. The addition of antifibrinolytic medications, including tranexamic acid or aminocaproic acid, can be helpful for those adolescent females with bleeding disorders or those adolescents in whom hormonal therapy is not desired.

### Adolescents with chronic health conditions

Many adolescents with chronic health conditions are at a higher risk of ID and its complications. These disorders include those that affect the gastrointestinal tract and heart failure.

#### Gastrointestinal disorders

Anemia in inflammatory bowel disease (IBD) is common and multifactorial. A quality improvement study that targeted screening patients with IBD aged <21 y for ID demonstrated that 88% of their cohort had either ID or iron deficiency anemia (IDA) [48]. ID anemia in patients with IBD occurs as a combination of gastrointestinal blood loss, intestinal malabsorption, and poor appetite [49]. In IBD, ID is often accompanied by anemia of inflammation or chronic disease, as with other inflammatory disorders, and differentiating between the 2 can be challenging. The inflammation from IBD induces hepcidin expression and production, decreasing the amount of bioavailable iron by preventing iron release from enterocytes and macrophages [50,51]. Although ID is common in adolescent females from HMB, an adolescent male presenting with ID should raise a red flag for potential blood loss secondary to gastrointestinal pathology.

#### Heart failure

In a retrospective cohort of children and adolescents with heart failure, ID was associated with an increased rate of adverse events (defined as ventricular assist device implantation, heart transplant, or death) compared with those patients who were iron-sufficient [52]. In adults with heart failure, ID has been associated with worsened health-related quality of life, regardless of the presence of anemia [53].

### Adolescent athletes

Individuals who engage in regular, intense exercise have increased iron losses compared with those who do not [7]. Male and female athletes have ≤1.75 and 2.3 mg/d iron losses compared with only 1.08 and 1.45 mg/d in nonathletes, respectively [54]. There are several mechanisms to account for this increased iron loss, including distance running-associated gastrointestinal blood loss, erythrocyte rupture in the foot during running, and training-associated inflammation resulting in decreased iron absorption and recycling [7,55,56]. Thus, adolescent athletes may have as much as 30% greater iron requirements compared to more sedentary adolescents [7].

Athletes training at high altitudes or hypoxic environments have higher hemoglobin concentrations, driven by an increase in erythropoietin production. Iron supplementation under these circumstances increased maximal oxygen consumption, whereas there was no improvement in those who train at sea level [57,58]. Runners at altitudes were calculated to require an extra 4.9 mg iron/d, whereas males and females runners at sea level require an additional 1.9 and 2.3 mg/d of iron, respectively [57].

Relative Energy Deficiency in Sport (RED-S), formerly referred to as the “female athlete triad,” refers to the combination of low energy that may or may not be related to disordered eating, menstrual dysfunction or amenorrhea, and low bone mineral density in physically active females [59,60]. This clinical scenario results from inadequate caloric energy relative to an individual’s energy expenditure and may also occur in males [61]. In high school-aged female athletes, the prevalence of 2 components of the triad was found to be as high as 18%. It is more common in individuals who participate in sports that emphasize endurance and aesthetics or have weight-class components [60]. ID has been hypothesized to play a role in bone loss secondary to its association with suppression of the growth hormone/IGF-1 axis [62]. Fibroblast growth factor 23 (FGF23) is also an important link between iron and calcium-phosphate homeostasis [63, 64].

### Adolescents with sleep and movement disorders

Many individuals with underlying neurologic or sleep disorders are ID and experience at least partial symptomatic improvement with iron therapy. ID in adolescents with neurodevelopmental disorders, including attention deficit hyperactivity disorder, may exacerbate the severity of their hyperactivity [65]. Those with autistic spectrum disorder are also at high risk of ID due to selective diet and food sensitivities [66]. Restless leg syndrome (RLS) is common, having a 2.0% prevalence in adolescence (12–17 y), and has an association with lower serum ferritin concentrations [67]. In a cross-sectional study of menstruating women, ferritin concentrations of ≤50μg/L and lower were associated with RLS [68]. A retrospective review in children with RLS and periodic limb movement disorder demonstrated that oral iron supplementation for individuals with ferritin values of <50 ng/ml had a sustained improvement in the periodic limb movement of sleep index for over 2 y [69].

## Oral Iron Supplementation

A large number of oral iron formulations are available both over the counter and online. Considered nutritional supplements rather than therapeutic drug preparations, many are available with limited data on efficacy. Iron salts, such as ferrous sulfate, ferrous fumarate, and ferrous gluconate, are the most established. Iron salts are in the ferrous (+2) form and are better absorbed than preparations in the ferric (+3) state. Ferric iron requires an acidic environment to ensure its reduction to the ferrous state for absorption.

Iron carbohydrates such as iron polysaccharide complex preparations are in the ferric form. They typically have improved palatability and are also effective but may require a longer duration of therapy due to less absorption. Carbonyl iron and other available forms of oral iron have limited efficacy data but can be effective and well tolerated.

### Dosing, frequency, timing

When considering iron supplements in adolescents, choosing a simple regimen that both maximizes absorption and is feasible within their schedule and lifestyle is imperative. The discovery of the hormone that regulates iron absorption, hepcidin [70], has informed how to best dose iron supplementation to achieve this goal [71]. Iron absorption studies in iron-deficient, nonanemic healthy women have demonstrated that morning doses of oral iron (>60 mg) caused an upregulation of hepcidin for ≤24 h [72]. During this time, iron absorption was impaired, and this effect increased with higher iron doses. To maximize iron absorption (specifically, the amount of iron in an individual dose), lower doses (single tablets containing 40–80 mg elemental iron daily) should be given rather than larger doses (e.g., 2 tablets of 65 mg elemental iron) [72]. Multiple daily doses should be avoided as there is limited benefit [72]. A follow-up study of nonanemic women with ID assessed iron absorption with administration of 14 doses of iron on either consecutive days (14 doses in 14 d) or alternate days (14 doses in 28 d), and the alternate-day dosing regimen resulted in 34% greater total iron absorption [73]. Again, it was found that splitting the dose (administering the same total amount of iron in 2 divided doses compared with 1 large dose) found no benefit. Similar studies of iron absorption in patients with moderate to severe IDA have not been performed. Based on this data, iron supplementation should be administered no more than once per day.

Hepcidin concentrations are lowest in the morning, presumably due to the fasting state in most individuals. Thus, morning doses of iron have higher absorption than those taken in the afternoon. Many adolescents ask about taking iron with foods to decrease gastrointestinal side effects. A recent study demonstrated that 100 mg iron consumption with coffee alone and breakfast decreases iron absorption by 54% and 66%, respectively [74]. The impact of coffee and tea ingestion on iron absorption is important to keep in mind for adolescents who have or are at risk of ID, especially those who are receiving iron supplementation.

For these reasons, the ideal supplementation regimen for adolescents is one tablet of ferrous sulfate taken in the morning with water or orange juice, separate from meals. Depending on the degree of IDA, daily (for those with anemia) compared with every other day (for those with ID alone) supplementation may be considered. Although dosing may be increased, administration should not occur more frequently than once daily. For adolescents in whom morning medication is not feasible, identifying the easiest time of the day to take the medication is key.

### Duration of supplementation

Total duration of iron supplementation is dependent on several factors and should be individualized based on both the adolescent’s laboratory response and types of symptoms experienced. For adolescents initiated on iron supplementation for anemia, iron supplementation should continue after resolution of the anemia to replenish the iron stores. The time it takes to replenish iron stores can vary based on whether the risk factors for ID have been fully addressed or remain present, but typically takes ≥2 to 3 mo to achieve. The simplest way to ensure the iron stores are restored is to check the ferritin value. For those otherwise healthy adolescents, a threshold of ferritin of 20 ng/mL should be achieved before discontinuing iron. In adolescents with persistent risk factors, ongoing intermittent supplementation may be necessary.

In adolescents experiencing symptomatic ID without anemia, higher ferritin thresholds may be utilized before stopping iron therapy. Specifically, individuals with fatigue or sleep and neurologic conditions, such as restless legs syndrome, may continue to have symptomatic improvement by achieving ferritin values of 50 ng/mL. In contrast, for iron-sufficient athletes, the practice of iron supplementation with the goal of increasing performance, often in endurance athletes, is controversial. Although improvements were noted in athletes with ferritin concentrations <30 ng/mL, currently, there is no recommended target ferritin for athletes above the normal set ferritin values [75]. Evaluation of higher goal ferritin concentrations in elite female athletes, targeting ferritin values of ≥100 ng/mL, has suggested that iron supplementation throughout a competitive season may be beneficial as iron stores are quickly depleted after cessation of supplementation [76].

### Intravenous iron

Although oral iron supplementation remains the mainstay of therapy for most adolescents with ID, in those who have an incomplete response (persistent ID), intravenous (IV) iron therapy is increasingly being used. The benefits of IV iron include the rapid repletion of iron stores in addition to the resolution of anemia, fewer gastrointestinal side effects, and relief for individuals struggling with long-term iron supplementation. Indications for IV iron as upfront therapy – without a trial of oral iron – are limited to adolescents with severe anemia and/or chronic conditions [77].

#### When to consider

The most common indication for IV iron in adolescents is failure to improve with oral iron [[78], [79], [80]]. Reasons for oral iron treatment failure are broad but include nonadherence or intolerance to oral iron [81], continued low-iron diet, suboptimal iron absorption, and/or ongoing uncontrolled blood loss experienced by adolescents with HMB or gastrointestinal disorders. General guidance for the recommended approach to iron replacement by patient type is demonstrated in Table 3.

**TABLE 3:** Guidance on iron replacement therapy in adolescents

Adolescent Group	Recommended Iron Supplementation	Other Considerations
Adolescents with iron deficiency anemia	Ferrous sulfate 1–2 tablets once daily in the morning with water or juice	Consider upfront intravenous iron in the setting of severe or symptomatic iron deficiency anemia
Adolescents with iron deficiency	Ferrous sulfate 1 tablet once daily or every other day in the morning with water or juice	Intravenous formulations may be considered if unable to replete iron stores after 3 to 6+ mo of oral iron secondary to poor compliance or intolerance
Adolescents with iron deficiency and inflammatory conditions	Referral for intravenous iron therapy	Ferritin may not be a reliable marker of their underlying iron status

The next most common indication occurs in patients with concomitant IDA and anemia resulting from inflammation who are therefore unable to absorb oral iron effectively [82]. In such patients, IV iron should be used. For those with IBD and ID, IV iron replenishment leads to increased resolution of the ID compared with oral replenishment. Adults with heart failure have also demonstrated improved clinical outcomes as well as health-related quality of life following IV iron therapy [83,84]. Data in children with heart failure have similarly found that oral iron may be ineffective compared with IV iron therapy with regards to resolution of IDA [85].

Adolescent girls and young women with fatigue or restless legs syndrome have been shown to benefit from IV iron therapy, particularly when their serum ferritin is <15 mg/L [86]. A small randomized, placebo-controlled study of adult patients with RLS syndrome demonstrated that a full treatment course of IV iron led to remission of clinically significant RLS for 25% of treated patients [87]. Retrospective studies that evaluated the effect of IV iron on children with RLS and periodic limb movement disorder demonstrated improvement in both clinical groups [69,88]. Administration of IV iron to distance runners with normal ferritin concentrations (between 30 and 90 ng/mL) did not result in improvements in physical performance though reported to improve mood and reduce fatigue [89].

#### Overview of available IV iron of formulations

Six IV iron formulations are available in the United States, 4 have approved indications in pediatrics, and many are increasingly used off-label in children as well [[78], [79], [80]]. As of 2024, the approved formulations include low-molecular-weight iron dextran, iron sucrose, ferric gluconate, and ferric carboxymaltose. Most pediatric data are available from retrospective case series and cohorts in diverse pediatric populations [90]. A case series of >500 infusions of iron sucrose in pediatric patients demonstrated an excellent response with very low rate of adverse events [78]. Low-molecular-weight iron dextran requires a test dose before full dose administration, but then can be administered in the form of total dose infusions in pediatric patients [79,91]. Total dose infusions allow for iron to be administered in a single infusion. Additional series have demonstrated safety and efficacy in children and adolescents receiving ferric carboxymaltose [80]. Although serious adverse events such as hypersensitivity and anaphylaxis are rare, all forms of IV iron therapy should be administered in a center with experience in its administration and where appropriate monitoring and anaphylactic precautions are provided [77].

## Discussion

Adolescents have unique physiologic needs that predispose them to being at risk of IDA. Low-iron diet related to common but poor eating habits in adolescents, high rates of disordered eating, as well as other risk factors, such as rapid growth and menstrual blood loss, are particularly important to consider. Dietary counseling that ensures appropriate sources of bioavailable iron, which is consistent with the adolescent’s preferred dietary practice (i.e., nonvegetarian, vegetarian, or vegan), is key to maintaining normal iron balance. In those adolescents who are iron-deficient, oral iron supplementation is recommended. Ferrous sulfate taken once daily or every other day in the morning with water or orange juice (without food) will optimize absorption. For adolescents with additional risk factors and/or medical conditions that complicate iron absorption, IV iron therapy may be considered and is supported by increased efficacy and safety data over the past decade.

### Authors’ contributions

The authors‘ responsibilities were as follows – CC and JP: contributed equally to the design, writing, and final content of the manuscript. Both authors have read and approved the final manuscript.

### Conflicts of interest

CC has no relevant conflicts of interest to disclose. JP previously served on an advisory board for Pharmacosmos LLC.

## Funding

The authors reported no funding received for this study.
